# Impact of prolonged storage time on homograft ultrastructures: an attempt to find optimal guidelines for homograft processing

**DOI:** 10.1007/s10561-024-10127-2

**Published:** 2024-02-22

**Authors:** Ida von Konow, Angeline Eliasson, Johan Nilsson, Torsten Malm

**Affiliations:** 1 Department of Cardiothoracic Surgery, Skane University Hospital, Lund, Sweden; 2 Department of Clinical Sciences, Thoracic Surgery, Lund University, Lund, Sweden; 3 Tissue Bank Lund, Skane University Hospital, Lund, Sweden; 4 National Board of Forensic Medicine, Lund, Sweden; 5 Department of Translational Medicine, Thoracic Surgery and Bioinformatics, Lund University, Lund, Sweden; 6 Department of Pediatric Cardiac Surgery Unit, Skane University Hospital, Lund, Sweden

**Keywords:** Homograft, Cardiovascular tissue, Cardiac surgery, Storage time, Tissue banking, Transmission electron microscopy

## Abstract

According to guidelines, total ischemic time for homografts at processing must be kept short to avoid degeneration. Many homografts are discarded due to practical inability to finish all steps from procurement to cryopreservation within the time limit. Although, several studies have shown that homografts with prolonged ischemic time show adequate quality and performance. Twenty aortic and 12 pulmonary homografts were collected and biopsies were retrieved at preparation (day 0) and after 1, 2, 3, 4, 7, 14, 21, 28, and 60 days in antibiotic decontamination at 4 °C. Biopsies were prepared for light microscopy (LM) and transmission electron microscopy (TEM). Assessment generated scores for cells, elastin, and collagen. Relative differences between times were compared with Wilcoxon signed rank test. Bonferroni corrected *p* value of 0.0056 was considered significant. LM could only reveal decrease in cell count at 60 days in aortic homografts, no other differences was detected. TEM showed affected cell appearance in day 3 and day 4 and beyond for aortic and pulmonary homografts respectively. Elastin appearance was affected at day 60 for aortic and day 21 for pulmonary homografts. Collagen appearance was affected at day 28 for aortic homografts, with no significant differences in pulmonary homografts. Cell degeneration starts early after homograft procurement, but elastic and collagen fibers are more resistant to degeneration. Overall structure integrity as seen in LM was not affected at all, while TEM could reveal small degeneration signs in individual elastic fibers and collagen bundles at 21 and 28 days respectively.

## Introduction

Cardiovascular homografts are one of the preferred heart valves used in reconstruction of the right ventricular outflow tract in congenital heart disease (Baumgartner et al. 2021). Long-term survival after implantation of homografts are excellent, but heart valve longevity is limited (Skoglund et al. 2017; Meijer et al. 2019; Romeo et al. 2019). Homografts are retrieved from human donor hearts, where tissue banks conduct processing and storage of these tissues.

To ensure homograft quality, European tissue banks adhere to guidelines developed from the European Directorate for the Quality of Medicine and Health Care (Doerr 2022). One important consideration in homograft processing is the maximum total ischemic time, i.e., time from donor circulatory arrest until cryopreservation of the homograft. Current guidelines recommend a maximum of 72 h of total ischemic time, with a maximum of six hours of warm ischemic time, and where at least 24 h must be allowed for antibiotic decontamination prior to cryopreservation. This leaves less than 48 h for the tissue bank to retrieve the tissue, transport it to the tissue bank, prepare the heart valves, and cryopreserve them after antibiotic decontamination. If strictly adhering to these guidelines, many homografts will be lost due to practical reasons (Botes et al. 2012).

Short ischemic time intend to keep homograft quality and prevent adverse events after implantation, but studies indicate that it would be safe to implant homografts with prolonged ischemic time (Meyns et al. 2005; Kalfa et al. 2011; Axelsson and Malm 2018; Bester et al. 2018). Looking at the homograft structure after prolonged ischemic time, it is shown that cellular viability decreases (Wallace et al. 1992; Niwaya et al. 1995; Gall et al. 1998). When the cell is exposed for starvation and degeneration, chromatin start to condensate within the cell nuclei (Burgoyne 1999), cell content starts to vacuolize and cells starts to shrink and detach from surrounding tissue (Wallace et al. 1992; Kristek et al. 1993).

Except for cells, the homograft is composed of extracellular matrix where elastin and collagen are the main components of the aorta and the pulmonary artery, providing strength and elasticity to the tissue (Burkert et al. 2021). Healthy vessels express lower content of collagen compared to vessels from older individuals and vessels exposed to hypertension, with intact elastic fibers (without fragmentation) and surrounding fibrillin to anchor mature elastin fibers to surrounding tissue (Kielty et al. 2002; Jordão et al. 2011; Halushka et al. 2016). Typical elastin degeneration signs are increased complexity, splitting, fragmentation and thinning of the fiber (Dingemans et al. 2006; Halushka et al. 2016). Collagen degeneration signs have previously been shown in vascular diseases such as aortic aneurysm and hypertension, and consist of increased waviness and disrupted fiber orientation, as well as disrupted D-bands with reduced contrast (Jones et al. 2020). So far, studies have shown that mechanical and morphological properties of the extracellular matrix of the homograft vessel walls and cusps seem to keep intact after prolonged cold ischemic time prior to organ harvest (Smit et al. 2015; Bester et al. 2018; Fabian et al. 2022). Forensic studies have shown that tissues with extracellular matrix that is rich in collagen and/or elastin, such as blood vessels, are resistant to both autolytic degeneration and microbiological decomposition and that adequate cooling slow the process even further (Cocariu et al. 2016; Javan et al. 2019).

The aim of this study was to investigate homograft tissue integrity and ultrastructures at different ischemic times, ranging from 0 to 60 days. The objective was to determine if it is possible to find an exact time point when the homograft starts to degenerate with signs of tissue integrity dissolution and quality impairment.

## Materials and methods

### Ethical statement

The Regional Ethical Review Board in Lund, Sweden, approved the study (Dnr 2017/133 and Dnr 2018/568).

### Homografts

Homografts were collected from the Tissue Bank in Lund during 2019–2022. Inclusion criteria were homografts that could not be used for transplantation due to structural impairment such as valve fenestrations, donor aged 18–65, and donors with donation after brain death (DBD). DBD donors were chosen to avoid impact from different ischemic times and warm ischemia prior to heart explantation, which is the case in non-heart beating donors. Atherosclerosis in the vessel wall was accepted if it was scarcely distributed, with sufficient healthy vessel left for biopsies.

Homografts were prepared according to regular routines. If structural impairment was found that excluded the homograft from transplantation, it was evaluated for inclusion in the study. If inclusion criteria were met, first biopsies were retrieved in conjunction with the preparation procedure, prior to antibiotic decontamination. Biopsies were retrieved from the vessel wall of the homograft and defined as “Day 0”. The homograft was then put in a regular antibiotic solution (200 ml Medium199 Earle’s salt, Vancomycin (100 mg), Gensumycin (106 mg) and Amphotericin B (50 mg)) and stored at 4 °C according to standard protocols. Additional biopsies were retrieved after 1, 2, 3, 4, 7, 14, 21, 28 and 60 days in the antibiotic solution. The full vessel wall of the homograft had to be used to be able to collect all biopsies (in aortic homografts including vessel just distal to the sinuses until the proximal aortic arch and in pulmonary homografts including vessel from just distal to the sinuses until the pulmonary bifurcation).

### Light microscopy

At each time, three biopsies with the size of 0.5 × 0.5 cm were retrieved from the homograft vessel wall. Samples were fixed in formalin at 4 °C directly after collection. Samples were dehydrated with increasing concentrations of ethanol and xylene and then embedded in paraffin under vacuum. Sections of 3,5 mm were placed on slides in heated distilled water and then dried overnight in a heating cabinet. Sections were stained with erythrosine saffron for inspection of cell nuclei, elastica van Gieson for elastin fibers and azan for collagen fibers. Samples were scanned with NanoZoomer-SQ Digital slide scanner (Hamamatsu Photonics, Hamamatsu-city, Japan) and assessed at a computer screen through NDP.view2 Image viewing software (Hamamatsu Photonics, Hamamatsu-city, Japan). Analysis was conducted with 20–40 × magnification.

### Transmission electron microscopy

At each time point, one biopsy with the size om 0,5 × 0,1 cm was retrieved from the homograft vessel wall. Biopsies were pre-fixed in 2% paraformaldehyde and 2% glutaraldehyde for 1–24 h and then rinsed in 0,1 Sorensen’s Phosphate Buffer. Samples were fixed with 1% OsO_4 _for one hour followed by dehydration with increasing concentrations of acetone. After dehydration, samples were impregnated and embedded in Epon, followed by polymerization in Epon for 48 h at 60 °C. Thin sections of 60 nm were sliced with Diatome diamond knife in Leica EM UC7 ultratome and sections were mounted on Maxtaform H5 formvar coated copper grid. Grids were contrasted with 4% uranylacetat for 20 min in 38 °C and 1% leadcitrate for 2 min in room temperature. Samples were examined with FEI Tecnai biotwin 120 kV microscope and images were obtained with Olympus veleta 2 × 2 k camera.

### Morphological interpretation

Light microscopy (LM) and transmission electron microscopy (TEM) samples were assessed separately with two different protocols. Cells, elastic fibers, and collagen fibers were analyzed separately in both modalities. Aortic and pulmonary homografts were analyzed in two separate groups.

#### Light microscopy

Evaluation of LM samples was blinded, the evaluator did not know which day the samples were from. One evaluator assessed all samples. A second evaluator assessed 20% of the samples to validate the protocol and check for inter-rater reliability.

**Fig. 1:**
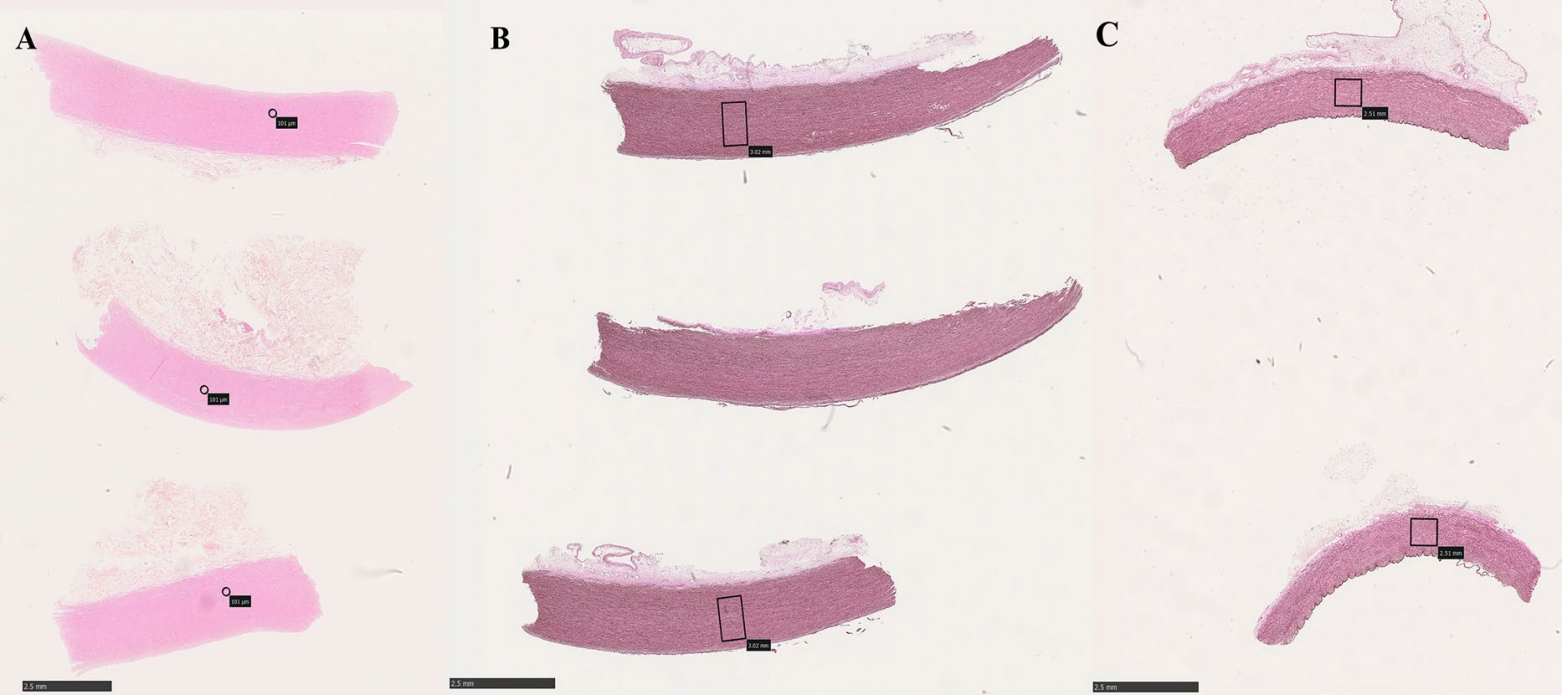
**A** Circles used for cell count and cell appearance evaluation. Erythrosine saffron stain, 0.87x magnification. **B** Boxes used for elastic fiber evaluation in aortic homografts. **C** Boxes used for elastic fiber evaluation in pulmonary homografts. Elastica van Gieson stain, 0.87 magnification

**Fig. 2:**
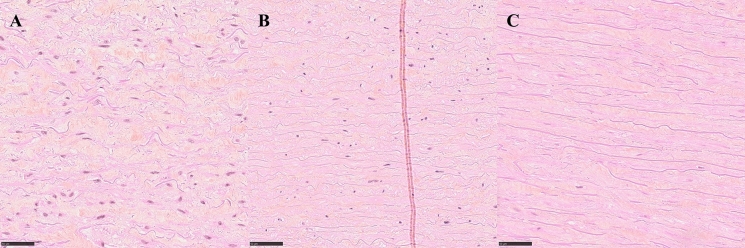
Typical examples of cell appearance. **A** Normal cells (day 2) with well-defined nuclei and chromatin. **B** Pyknotic cells (day 2) with small, homogenous nuclei. **C** Karyolotic cells (day 60) with dissolving nuclei. Erythrosine saffron stain. 40 × magnification. Scale bar 50 μm

Cells were evaluated with two different categories, cell count and cell nuclei appearance. Three circles with a radius of 100 µm were placed in the media of the vessel wall, one close to the endothelial area, one in the middle of the media and one close to the adventitial area. Cell count was calculated as a continuous variable, by counting total cell count within each circle (Fig. 1A). Cell nuclei appearance was evaluated within the defined circles as well. Cell nuclei was defined as normal, pyknotic or karyolitic in each circle, and given separate scores of 1–3 depending on the dominated cell type (Fig. 2) (Table 1).

**Table 1:** Summary of evaluation protocol for light microscopy

Structure	Description
**Cell Count**	The total amount of cells found in all three circles
**Cell nuclei appearance**	
Score 3	Normal cell nuclei with well-defined nuclei membrane
Score 2	Pyknotic cells with small, dark, homogenous nuclei
Score 1	Karyolitic cells with dissolving nuclei and diffuse nuclei membrane
**Elastin appearance** (aortic/pulmonary)	Each intact elastin fiber > 100 µm for aortic homografts and > 50 µm for pulmonary homografts was given a separate point. Longer fibers generated higher points. Total points were calculated in each defined area
> 100/50 µm	1 point each
> 150/100 µm	2 points each
> 200/150 µm	3 points each
> 250/200 µm	4 points each
**Collagen appearance**	
Score 5	Well defined collagen bundles, with > 5 bundles of 75 µm length
Score 4	Well defined collagen bundles, but shorter and more fragmented
Score 3	Less defined bundles that are starting to dissolve
Score 2	Ill-defined bundles and dissolved fibers, but still intact pattern of collagen withing the sample
Score 1	No visible collagen bundles, no well-defined pattern of collagen within the sample

Elastin was evaluated as a continuous variable, counting the number of intact elastin fibers. Two areas of 0.8 mm^2^ (aortic) or 0.4 mm^2^ (pulmonary) were chosen at random for each sample. Smaller area was chosen for pulmonary homografts since their vessel wall was thinner. Within the area, all intact elastin fibers > 100 µm (aortic) or > 50 µm (pulmonary) were counted (Fig. 1B-C). Different lengths were chosen since the normal aorta express more and longer elastic fibers compared to the normal pulmonary artery. Longer fibers were given a higher score (Table 1).

**Fig. 3:**
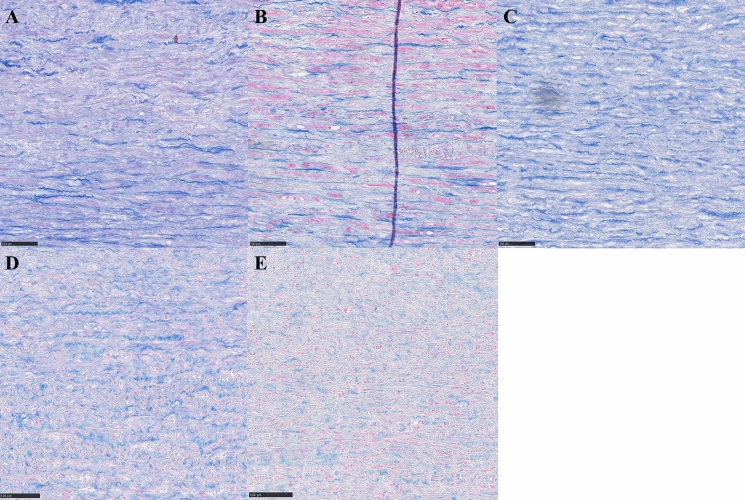
Typical examples of collagen appearance. **A** Well-defined, long collagen bundles (day 2), score 5. **B** Well-defined, but shorter collagen bundles (day 3), score 4. **C** Still visible bundles, but starting to dissolve (day 2), score 3. **D** Ill-defined bundled with dissolving fibers (day 21), score 2. **E** Collagen pattern completely dissolved, no visible bundles (day 60), score 1. Azan stain, 20 × magnification. Scale bar 100 μm

Collagen was evaluated as a categorical variable. Two separate areas of the sample were evaluated, and each area was given a score from 5 (normal) to 1 (complete destruction) (Table 1) (Fig. 3).

A summary of the evaluation protocol is shown in Table 1.

#### Transmission electron microscopy

TEM was used to evaluate ultrastructures of the homografts. The evaluation was not blinded. One evaluator assessed all samples. Five cells, five elastin fibers and five areas with collagen were inspected and evaluated separately within each sample.

**Fig. 4:**
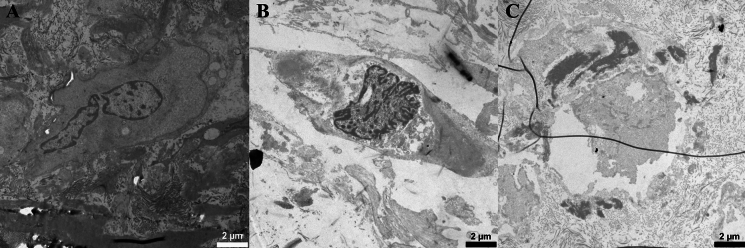
Typical examples of cells in transmission electron microscopy. **A** Non-condensed, well scattered chromatin (score 4) and no cell shrinkage (score 5) (day 0, 6000 × magnification). **B** Almost fully condensed chromatin appearing as lumps within the nuclei (score 2) and cell shrinkage with presence of vacuoles (score 1) (day 14, 6000 × magnification). **C** Completely dissolved nuclei (score 1) with no distinguished chromatin (score 1) and cell shrinkage (score 2) (day 60, 6000 × magnification)

**Fig. 5:**
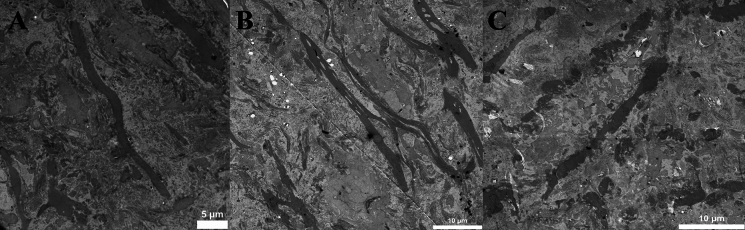
Typical examples of elastin in transmission electron microscopy. For assessment of fibrillin, a higher magnification was used. **A** Well-defined fiber border without damage (score 5), no internal damage (score 3) (day 3, 2550 × magnification). **B** Partly defined and partly uneven border (score 4), multiple, large areas with internal damage (score 1) (day 4, 6000 × magnification). **C** Partly uneven border, partly dissolving (score 2), small internal damage (score 2) (day 21, 6000 × magnification)

**Fig. 6:**
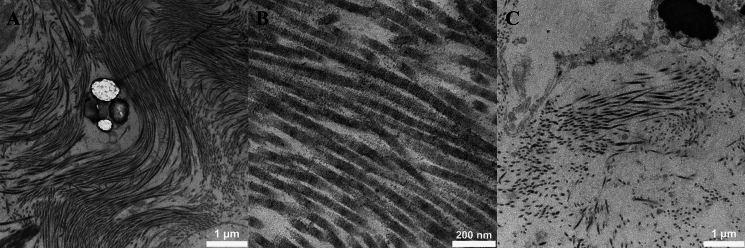
Typical examples of collagen in transmission electron microscopy. **A** Dense bundle with parallel fibers (score 3), no shrinkage of bundles (score 2) (day 3, 16,500 × magnification). **B** Clear presence of D-banding pattern (score 3) (day 28, 87,000 × magnification). **C** Sparse and disoriented fibers within the collagen bundle (score 1), shrinkage of bundle with loss from surrounding tissue (score 1) (day 60, 16,500 × magnification)

Cell appearance was evaluated by chromatin appearance, cell nuclei membrane and cell shrinkage (Fig. 4). Elastin appearance was evaluated by fiber structure and fragmentation, damage within the fibers and presence of surrounding fibrillin (Fig. 5). Collagen appearance was evaluated by collagen bundle structure and orientation, shrinkage of bundles and presence of typical D-banding pattern (Fig. 6). A summary of the evaluation protocol is shown in Table 2.

**Table 2:** Summary of evaluation protocol for transmission electron microscopy

Structure	Description		
**Cell appearance (total score 3–12)**	Cell nuclei membrane appearance (score 1–5)	Chromatin appearance (score 1–4)	Cell shrinkage (score 1–3)
Score 5	Well defined, double membranes visible		
Score 4	Well defined, double membrane only partly visible	Non-condensed chromatin, well scattered within the cell nuclei	
Score 3	Well defined, but only single membrane visible	Partly condensed chromatin, still scattered within the cell nuclei	No cell shrinkage
Score 2	Partly defined, partly dissolved	Fully condensed chromatin, appearing as lumps within the nuclei	Cell shrinkage, loss from surrounding tissue
Score 1	Completely dissolved	Complete dissolved chromatin that cannot be distinguished	Cell shrinkage and presence of vacuoles
**Elastin appearance (total score 3–10)**	Elastin fiber structure (score 1–5)	Elastin fiber internal damage (score 1–3)	Presence of surrounding fibrillin (score 1–2)
Score 5	Well defined fiber border without damage		
Score 4	Partly defined fiber border, partly uneven and ruffled		
Score 3	Uneven, ruffled fiber border	No internal damage	
Score 2	Partly uneven fiber border, partly dissolving	Smaller damage, 2 or less areas per field of view at 6000 × magnification	Presence of surrounding fibrillin
Score 1	Dissolving fiber	Single, large damage or multiple areas with damage	No surrounding fibrillin
**Collagen appearance (total score 3–8)**	Collagen bundle structure and orientation (score 1–3)	Presence of typical D-banding of fibers	Shrinkage of bundles (score 1–2)
Score 3	Dense bundles with parallel fibers	Clear presence of D-banding	
Score 2	Dense bundles but disorientation of fibers within the bundles	Partly dissolved D-banding	No shrinkage, bundles lie adjacent to surrounding tissue
Score 1	Sparse and disoriented fibers within the collagen bundle	No D-banding	Shrinkage of bundles, loss from surrounding tissue

### Statistical analysis

Results are presented as medians with interquartile range.

Differences in score from morphological analysis was analyzed with Wilcoxon signed rank test for paired data. For LM, day 0 was used as reference day and all other groups were compared separately to the result from day 0. If day 0 was missing, day 1 was used as reference day. Score at day 0 were set to 1, and relative difference between groups were calculated, to minimize impact by differences between different homografts. For TEM, day 1 was used as reference day. Due to practical issues, proper material for TEM (formaldehyde) could not be retrieved at day 0 for all samples. To minimize impact from missing data, day 1 was used as reference day instead. If both day 0 and day 1 was missing, day 2 was used as reference day. Post-hoc test for multiple comparisons were conducted with the Bonferroni correction.

Correlation between the result from the two different evaluators in LM were analyzed with intraclass correlation coefficient (ICC) for continuous variables and weighted Cohen’s Kappa for categorical, ordinal variables.

Analyses were performed with Stata Statistical Software (StataCorp. 2017. Release 15. College Station, TX: StataCorp LLC, USA).

Main outcome was relative differences in score for cells, elastin and collagen between the reference day and longer time intervals.

## Results

### Homografts

Donor and homograft characteristics are presented in Table 3. Since all donors were DBDs, there was no ischemic time prior to explantation. Explantation to preparation time is defined as time from heart explantation until homograft procurement in the tissue bank. During this period the heart is stored in a cold environment, without antibiotic treatment. Only one aortic and one pulmonary homograft had an explantation to preparation time that exceeded 48 h.

**Table 3:** Donor and homograft characteristics

**Variable**	Aortic (n = 20)	Pulmonary (n = 12)
**Donor age**, median (range)	53 (24–65)	51.5 (40–64)
**Donor gender**		
Female Male	9 (45%) 11 (55%)	8 (66.7%) 4 (33.3%)
**Discard reason**		
Fenestration Atheromatosis Valve leakage Preparation damage Unknown Vegetation on cusp	9 (45%) 7 (35%) 2 (10%) 1 (5%) 1 (5%)	11 (91.7%) 1 (8.33%)
**Heart collection to preparation**, hours (median, range)	36.5 (5–58)	35 (10–58)

### Morphological interpretation

Each category was analyzed nine different times (each time point compared separately to the reference time point), resulting in a Bonferroni corrected *p* value of 0.0056.

#### Light microscopy

Each homograft (n = 32) generated biopsies from 10 different times. Sample from day 0 was missing in two aortic homografts. Sample from day 60 was missing in one pulmonary homograft. In total, 317 samples were analyzed, and 60 samples (19%) were randomly selected for blinded evaluation by an additional evaluator to assess validity of the protocol. Result from analysis of inter-rater reliability is shown in Table 4. All four categories have a correlation between 75 and 95%, considered substantial to almost perfect agreement according to Landis and Koch (1977).

**Table 4:** Inter-rater reliability calculated with intraclass correlations for continuous variables and weighted Cohen’s Kappa for ordinal categorical variables

	Intraclass correlation	Cohen’s Kappa agreement	CI (95%)
Cell count	95%		91–97%
Cell appearance		75%	68–83%
Elastin appearance	89%		82–93%
Collagen appearance		86%	78–93%

Result from evaluation of samples in LM are shown in Table 5 for aortic homografts and Table 6 for pulmonary homografts. When using Bonferroni corrected p-value, only cell count showed a significant difference when comparing day 0 to day 60 in aortic homografts.

**Table 5:** Result from light microscopy evaluation of aortic homografts. Day 0 is used as reference. Relative differences are calculated. * = significant with Bonferroni corrected *p* value (0.0056)

	Cell count			Cell appearance			Elastin appearance			Collagen appearance		
Day	Median	IQR	*p* value	Median	IQR	*p* value	Median	IQR	*p* value	Median	IQR	*p* value
0	1.0			1.0			1.0			1.0		
1	0.91	0.68–1.4	0.90	1.0	0.66–1.5	0.40	1.0	0.71–1.9	0.49	1.0	0.82–1.2	0.59
2	1.03	0.69–1.2	0.77	1.0	0.67–1.3	0.81	0.62	0.37–2.0	0.96	1.0	0.73–1.1	0.38
3	0.85	0.70–1.1	0.31	0.79	0.63–1.3	0.18	1.4	0.76–3.0	0.059	1.0	0.75–1.3	0.57
4	0.90	0.66–1.2	0.33	0.94	0.67–1.3	0.51	0.61	0.17–1.7	0.65	1.1	0.83–1.5	0.18
7	0.95	0.82–1.2	0.61	0.85	0.67–1.1	0.29	1.1	0.47–1.8	0.50	1.0	0.73–1.2	0.65
14	0.91	0.64–1.6	0.48	0.92	0.56–1.4	0.69	0.64	0.38–1.2	0.32	0.94	0.66–1.5	0.88
21	0.98	0.57–1.3	0.91	0.75	0.6–1.3	0.24	0.56	0.38–1.2	0.22	1.0	0.64–1.2	0.40
28	0.90	0.54–1.3	0.32	0.67	0.5–1.2	0.12	1.1	0.20–2.1	0.36	1.0	0.80–1.3	0.97
60	0.57	0.51–0.97	0.0019*	0.80	0.50–1.0	0.0070	0.75	0.38–1.5	0.72	0.93	0.80–1.2	0.71

**Table 6:** Result from light microscopy evaluation of pulmonary homografts. Day 0 is used as reference. Relative differences are calculated

	Cell count			Cell appearance			Elastin appearance			Collagen appearance		
Day	Median	IQR	*p* value	Median	IQR	*p* value	Median	IQR	*p* value	Median	IQR	*p* value
0	1.0			1.0			1.0			1.0		
1	0.79	0.63–1.0	0.041	1.0	1.0–1.3	0.083	0.86	0.52–1.5	0.81	1.2	1.0–1.7	0.049
2	0.80	0.60–0.91	0.10	1.1	0.90–1.3	0.23	1.2	0.83–2.3	0.22	1.2	0.92–1.6	0.064
3	0.88	0.49–1.0	0.071	1.2	0.90–1.3	0.15	1.0	0.83–1.7	0.41	1.3	1.0–1.75	0.022
4	0.92	0.75.1.1	0.48	1.1	1.0–1.5	0.096	1.0	0.48–1.3	0.75	1.0	0.57–1.75	0.61
7	0.83	0.52–1.03	0.071	1.0	0.93–1.1	0.65	1.1	0.64–2.7	0.25	1.1	1.0–1.5	0.077
14	0.74	0.63–1.5	0.91	0.89	0.83–1.1	0.45	1.1	0.50–1.5	0.61	1.0	0.78–1.1	0.52
21	0.88	0.61–1.5	0.94	0.93	0.73–1.1	0.69	1.0	0.65–1.2	0.75	1.0	0.93–1.3	0.49
28	0.97	0.57–1.3	0.70	0.65	0.53–1.0	0.090	1.1	0.75–2.1	0.15	1.2	0.92–2.0	0.17
60	0.67	0.51–1.2	0.21	0.60	0.50–0.78	0.029	0.92	0.43–1.3	0.56	1.3	1.0–1.5	0.045

#### Transmission electron microscopy

Samples from day 0 was missing from 11 aortic and seven pulmonary homografts. Samples from day 1 was missing from two aortic and one pulmonary homograft. Samples from day 21 was missing from one aortic and one pulmonary homograft. Samples from day 60 was missing from one pulmonary homograft. In total, 296 samples were analyzed.

Result from evaluation of samples in TEM are shown in Table 7 for aortic homografts and Table 8 for pulmonary homografts. For aortic homografts, all samples from day 3 and beyond showed significantly more cell degeneration compared to day 1. For pulmonary homografts, all samples from day 4 and beyond showed significantly more cell degeneration compared to day 1. Elastin appearance show significantly more degeneration at day 60 for aortic homografts and at day 21 for pulmonary homografts. Collagen appearance show significantly more degeneration at day 28 for aortic homografts. No significant differences in collagen appearance are shown in pulmonary homografts.

**Table 7:** Result from transmission electron microscopy evaluation of aortic homografts. Day 1 is used as reference. Relative differences are calculated. * = significant with Bonferroni corrected *p* value (0.0056)

	Cell appearance			Elastin appearance			Collagen appearance		
Day	Median	IQR	*p* value	Median	IQR	*p* value	Median	IQR	*p* value
0	1.0	0.98–1.1	0.44	0.95	0.95–1.2	1.0	1.0	1.0–1.1	0.34
1	1.0			1.0			1.0		
2	0.95	0.89–1.0	0.013	0.95	0.92–1.9	0.14	1.0	0.89–1.1	0.78
3	0.88	0.81–0.92	< 0.001*	1.0	0.90–1.1	0.75	1.0	0.91–1.1	0.99
4	0.84	0.77–0.93	< 0.001*	0.95	0.90–1.1	0.20	1.0	0.95–1.1	0.82
7	0.82	0.77–0.89	< 0.001*	0.97	0.90–1.0	0.30	0.96	0.80–1.1	0.38
14	0.67	0.62–0.78	< 0.001*	0.90	0.83–1.0	0.025	0.98	0.84–1.1	0.47
21	0.67	0.63–0.76	< 0.001*	0.92	0.80–1.0	0.073	0.93	0.85–1.0	0.23
28	0.64	0.55–0.68	< 0.001*	0.90	0.82–0.99	0.014	0.86	0.80–0.93	0.0013*
60	0.53	0.48–0.58	< 0.001*	0.90	0.83–0.99	0.0051*	0.95	0.90–1.1	0.57

**Table 8:** Result from transmission electron microscopy evaluation of pulmonary homografts. Day 1 is used as reference. Relative differences are calculated. * = significant with Bonferroni corrected *p* value

	Cell appearance			Elastin appearance			Collagen appearance		
Day	Median	IQR	p value	Median	IQR	p value	Median	IQR	p value
0	1.1	1.0–1.2	0.14	1.0	0.89–1.1	0.89	1.0	0.96–1.2	0.59
1	1.0			1.0			1.0		
2	0.98	0.94–1.0	0.46	1.0	0.94–1.1	0.61	1.0	0.87–1.1	0.91
3	0.93	0.87–0.99	0.041	0.97	0.93–1.0	0.31	1.0	0.93–1.2	0.35
4	0.87	0.78–0.94	0.0037*	0.97	0.88–1.0	0.084	1.1	0.89–1.1	0.94
7	0.78	0.76–0.86	0.0022*	0.89	0.85–1.0	0.028	1.0	0.76–1.1	0.31
14	0.78	0.69–0.93	0.0022*	0.99	0.91–1.1	0.78	0.90	0.81–0.97	0.054
21	0.68	0.53–0.73	0.0033*	0.91	0.82–0.97	0.0049*	1.0	0.84–1.1	0.86
28	0.71	0.67–0.79	0.0022*	0.97	0.94–1.0	0.41	1.0	0.81–1.1	0.97
60	0.55	0.49–0.71	0.0033*	0.99	0.91–1.0	0.31	0.91	0.84–0.97	0.11

## Discussion

This study investigates the presence of autolytic changes in homografts with prolonged cold ischemic time during antibiotic decontamination. It is the first study to investigate the homograft at a total of ten different time points during this step of the homograft processing process. Current guidelines recommend a maximum of 72 h of total ischemic time. This guideline is based on opinion and experience only, although repeated studies have shown good performance of homografts with prolonged total ischemic times (Meyns et al. 2005; Kalfa et al. 2011; Axelsson and Malm 2018; Bester et al. 2018).

In LM, it was difficult to detect any major degeneration of either cells, elastin fibers, or collagen fibers, showing that the overall structural integrity is well preserved at prolonged decontamination time. The only significant difference that could be found was a decrease in cell count at 60 days in aortic homografts. When looking at TEM samples, cells start to degenerate after 3–4 days of antibiotic decontamination with increased condensation of chromatin, dissolving cell nuclei membrane and detachment from surrounding tissue, all findings that can be difficult to detect with LM alone. Other studies confirm that cell viability decrease early, especially in warm ischemic environments (Livi et al. 1987; Niwaya et al. 1995; Gall et al. 1998). Wallace et. al (1992) describe that after 36 h of warm ischemic time, about 30% of the cells show irreversible cell injury. Similar signs of cellular injury can be seen in vascular diseases such as hypertension and atherosclerosis (Okruhlicová et al. 2008), indicating that viable cells could be important for vessel wall function in homografts. Niwaya et al. (1995) could show a negative correlation between cell viability and warm ischemic time. The importance of cell viability for optimal homograft function is not completely clear, but many studies indicate that the recipient will re-populate the tissue if no donor cells are present. Yu et al. (2011) implanted homografts into dogs and could show that after 12 months of implantation, the endothelial surface of the homograft was covered by the recipient’s own cells. Viable fibroblasts could be observed as well, but it was not possible to determine if they were of donor or recipient origin. Hazekamp et. al (1995) have shown that homograft implanted in pigs, explanted after 3–4 months, showed presence of both recipient and donor cells. Mitchell et al. (1995) analyzed explanted homografts from humans (8 h to 9 years after implantation) and could show that homografts implanted for a longer period expressed almost no cellular viability, but a preserved collagen network of donor origin. Studies also suggest that viable donor cells in the homograft could start an immune response in the recipient, which could speed up degeneration of the tissue (Welters et al. 2001; Shaddy and Hawkins 2002). This suggestion is also supported by studies showing promising results when using completely decellularized homografts (Boethig et al. 2019). When implanting decellularized homografts into pigs and explanting them after 15 months, investigation could show population of cells in both vessel and cusps with reendothelialization of the aorta and cusps surfaces and production of new collagen and elastin in the tissue (Gallo et al. 2016). Initial 5- and 10 year follow-up of decellularized homograft implantation in humans have shown promising results, indicating that the recipient does not depend on viable cells from the donor to ensure homograft function (Boethig et al. 2019). Up to this day, there are no clear consensus on whether cell viability is crucial for homograft longevity, but the good performance of completely decellularized homografts indicate that it is not necessary to strive for viable cells prior to cryopreservation.

Degeneration of collagen and elastin structures was less compared to cells. LM analysis could not show any degeneration of elastin or collagen for up to 60 days of cold ischemic time in the antibiotic solution, with preserved tissue structure and integrity. TEM could detect small differences, with detectable collagen degeneration at 28 days in aortic homograft and elastin degeneration at 60 days in aortic and 21 days in pulmonary homografts. It is known that these proteins are resistant to autolytic degeneration which is confirmed by our results (Cocariu et al. 2016; Javan et al. 2019). Similar findings have been demonstrated by others. Livi et al. (1987) investigated 20 homografts (aortic and pulmonary) after 2 and 4 weeks of cold storage in an antibiotic solution and could not see any structural differences in LM compared to the day of harvest. Fabian et al. (2022) investigated 57 homografts prior to implantation, where a majority of samples showed a mild degree of structural degeneration that correlated to higher donor age. However, degeneration was not correlated to prolonged cold ischemic time prior to antibiotic decontamination (12–21 h), neither prolonged antibiotic decontamination (12–21 days). Correlation between degeneration and donor age is well known from the literature (Halushka et al. 2016). Forensic studies of collagen in gingival tissue show that prolonged warm ischemic time (from 2 to 9 days) show a gradual degeneration of tissue with progressive disorientation of collagen bundles, indicating that this in the natural course for autolytic changes in collagen. Although, this process is significantly slowed down if the tissue is cooled (Mazzotti et al. 2019) and signs of collagen disorientation could not be seen until at least 28 days of cold storage in our material. While cells tend to degenerate early, elastin and collagen seem to keep its structural integrity for at least 21 days in cold storage. Still, early signs of collagen and elastin degeneration is subtle, and seem to be difficult to detect with LM alone.

## Limitations

Literature describes that previous medical history and increased age has significant impact of the extracellular proteins of the vessel wall (Halushka et al. 2016). Our material had a heterogenous group of donors which could impact the result. Due to limited availability of tissue, especially for pulmonary homografts, the investigated groups were small.

## Conclusion

Our material shows evidence of cell degeneration after a total of 3 days of antibiotic decontamination in a cold environment, preceded by a median cold ischemic period of 35–37 h (pulmonary vs. aortic homografts) prior to homograft preparation. Earliest signs of elastin and collagen degeneration could be found after 21 and 28 days respectively, showing high resistance to degenerative processes. Overall structural integrity as seen in LM was not affected at all at 60 days, only TEM could reveal small degeneration signs in individual elastic fibers and collagen bundles. We suggest that homograft decontamination in a cold environment could be prolonged to at least 14 days without affecting the quality of tissue matrix.

## Data availability statement

Data is available upon request.

## Conflict of interest

The authors have no competing interests to declare that are relevant to the content of this article.

## Abbreviations

DBD: Donation after brain death
LM: Light microscopy
TEM: Transmission electron microscopy
